# Exploring the relationship between context and obsessions in individuals with obsessive-compulsive disorder symptoms: a narrative review

**DOI:** 10.3389/fpsyt.2024.1353962

**Published:** 2024-02-14

**Authors:** Franziska Weiss, Kristina Schwarz, Tanja Endrass

**Affiliations:** Chair of Addiction Research, Institute of Clinical Psychology and Psychotherapy, Technische Universität Dresden, Dresden, Germany

**Keywords:** obsessive-compulsive disorder, self-report, obsessive behavior, cognition, mental health

## Abstract

Obsessions in obsessive-compulsive disorder (OCD) have long been proposed to differ from intrusive thoughts in unaffected individuals based on appraisal of the thoughts. However, more recent research indicates that cognitive processes behind obsessions may differ significantly from those in healthy individuals concerning their contextual relationship. This narrative literature review summarizes current evidence for the role of context-relatedness for obsessions in OCD and intrusive thoughts in affected and unaffected individuals. The review encompasses a total of five studies, two of which include individuals diagnosed with OCD (one study also includes a group of unaffected control individuals), while the other three studies investigate the relationship between OCD symptoms and context in unaffected individuals. As assessed by mainly self-reports, the review examines the connection between thoughts and their context, shedding light on how the repetition and automaticity of thoughts, as well as their detachment from context over time contribute to defining obsessions in contrast to intrusive thoughts. However, the link with context depends on the content of the obsessions. We propose the term “decontextualization of thoughts” to describe the phenomenon that obsessions gradually lose their connection with external context during the development of OCD. Future research should investigate whether this hypothesis can be supported by experimental evidence and identify whether this shift might be more likely a cause or a consequence of the disorder.

## 1 Introduction

Obsessive-compulsive disorder (OCD) is a serious and common mental disorder with a lifetime prevalence of about 2.3% (1). Despite recent increases in prevalence due to the COVID-19 pandemic (2, 3), OCD is considered a “neglected mental disorder” that attracts comparatively little public attention (4) and receives less than 1% of the total amount of mental health research funding (5). The disorder is characterized by obsessions, which are defined as recurrent intrusive thoughts, impulses or images. Obsessions lead to subsequent compulsions, which are actions taken to alleviate the distressing thoughts or to prevent negative outcomes associated with them (6). Obsessions and compulsions as the core symptoms of OCD have been well-studied and frequent themes include contamination, checking and symmetry (7–9). Appraisal of intrusive thoughts has been found to significantly predict OCD severity (10) and is an important aspect of cognitive models of OCD. Although there is some overlap in the content between intrusive thoughts in healthy participants and obsessions in OCD (11, 12), obsessions are often ego-dystonic, unwanted and persist over time (6). However, there is limited understanding of the specific triggers or context-related factors that contribute to the development of obsessions. It has been proposed that differences may exist in the contextual factors that influence the occurrence of intrusive thoughts and obsessions, particularly in terms of their proximity to the present situation or context (12–14).

Early research suggests that intrusive thoughts in healthy individuals usually have identifiable external or internal triggers, but obsessions in OCD are just as likely to be internally or externally triggered as they occur with no discernible trigger at all (11). As an example, the obsession of harming a child could be triggered by external stimuli, like the sight of a child or objects that could potentially be used to harm a child (11). Thus, intrusive thoughts and obsessions can either occur spontaneously or be externally triggered, with the former category usually causing more distress (15). In line, Lee and Kwon (16) proposed a distinction between two types of obsessions, each with distinct characteristics. Autogenous obsessions occur spontaneously and include sexual, aggressive and immoral thoughts or impulses. In contrast, reactive obsessions are triggered by specific stimuli within the context and include thoughts about contamination, mistake, accident, asymmetry, and loss. The weaker link between obsessions in OCD and their context as based on early findings (11) has been addressed in a more recent study. This study showed a greater tendency for obsessions to be indirectly rather than directly linked to context as a characteristic feature of OCD, distinguishing it from healthy control participants (12). Additionally, it has been demonstrated that nonclinical individuals with a higher frequency of context-unrelated thoughts tend to exhibit increased OCD symptoms (13). These results suggest that the cognitive processes underlying obsessions may significantly deviate from those in healthy individuals, particularly in terms of their contextual relationship (13). Obsessions in OCD appear to exhibit a weaker connection to the context, and this relationship may further diminish as the disease progresses.

Context is defined as the sum of circumstances that characterizes the experience of the current situation (17). It serves to characterize the situation and synthesizes all internal (such as physiological states, cognitive, emotional, biographical history) and external (including environmental and social) aspects of a situation (17, 18). In terms of the regulation of thoughts, it is adaptive to align them with the environment or external context. For example, thinking about last nights dinner is more useful when trying to recreate the same meal, than when trying to steer the car out of a tight parking lot.

The alignment of obsessions in OCD with their context of occurrence can thus be examined at two different levels: the internal and the external context (13, 19). The internal context originates from within the person, while the external context can be perceived and assessed through the senses, including sight, hearing, smell, taste, and touch (13, 19). If an obsession matches the internal context, it matches the individuals ongoing thought processes, emotional state or the biographical history (13). An obsession that matches with external context is one in which its content is directly linkable to an event that is monitored with the senses (12). Certainly, both are often interconnected. In addition, certain obsessions, such as the thought of harming ones own child, may have connections to the external context but strongly contradict the individuals internal beliefs and values. Following this, greater adaptability arises when thoughts harmonize with the context, facilitating a more integrated mode of action. Conversely, context incongruent thoughts, which are unexpected or even in conflict with internal beliefs and values, generate larger prediction errors and surprise signals, which may contribute to the overinterpretation of thoughts, thereby exacerbating OCD. Furthermore, incongruent thoughts likely distract from the task at hand and lead to decreased performance. The primary objective of this narrative review is to examine and summarize the available research concerning the role of context and context-relatedness of obsessions in OCD. The literature search was conducted with the search terms (“obsessions” or “OCD”) and context in the search engine Google Scholar. Results were manually screened for relevance and literature references in relevant articles were further screened for relevant articles.

## 2 Obsessions and their relationship with context

In this section, we will present studies that specifically explore the role of context in the manifestation of obsessions. Our review encompasses a total of five studies that investigate the interplay between thoughts and their context in OCD symptomatology. Of these, two studies were conducted in individuals diagnosed with OCD (12, 14), one of them also including a group of unaffected control individuals, while three studies focused on OCD symptoms in unaffected participants (13, 19, 20). Recognizing the heightened significance of the two clinical studies in elucidating the role of context in OCD, we will initiate our discussion by presenting these. Short summaries of the included studies can be found in **Table 1**.

**Table 1:** Information on the five main studies described.

References	Study population	Design and measures	Selected outcomes reported
Julien et al. (12),	N = 33 individuals with OCD, N = 90 non-clinical participants	**Measure:** Intrusion Inventory II: self-report and expert ratings. Part A: Occurrence of intrusions. Report frequency of each of 68 intrusions. Part B: Context of occurrence. Selected three most disturbing intrusions experienced in life, indicate context link of these (direct, indirect, no link). Context link compared between groups.	↑ intrusions and distress in individuals with OCD compared to non-clinical participants (HC) ** Context**: Proportion of direct and indirect links reversed in the two groups: • Direct links: OCD < HC (1/3 vs. > 1/2) • Indirect links: OCD > HC (1/2 vs. > 1/3 • No link: OCD > HC (16% vs. 8%) **Context and content**: Context link differed significantly depending on the content of intrusions. • More direct links to context: symmetry, hoarding • More indirect links to context: rumination, washing
Audet et al. (19),	N = 248 non-clinical participants	**Measures**: Vancouver Obsessional Compulsive Inventory (VOCI; 21), Fear of Self Questionnaire (FSQ; 22), Ego Dystonicity Questionnaire (EDQ; 23), Inferential Confusion Questionnaire Expanded Version (ICQ-EV; 80); Obsessive Beliefs Questionnaire Threat, Responsibility, Importance and control of thoughts, Perfectionism/uncertainty (OBQ-TRIP; 24) **Report** an intrusive thought plus two items from Personal Significance Scale (25): 1. “What caused these thoughts to occur when they started?”, 2. “Why do you think these thoughts keep coming back?” (assessment of context) **Clinicians rating** on 1) content (i.e. checking/contamination), 2) ego-dystonicity (yes/no), 3) evidence (with direct evidence/no direct evidence), 4) OCD-Relevancy (yes/no)	Frequencies: • Intrusive thoughts **contents:** unacceptable thoughts and impulses (46.4%), contamination (6.5%), checking (15.4%), other (28.2%) • **Context links**: without evidence (68.9%), with direct evidence (26.6%) • Ego-syntonic (61.9%), ego-dyston (32%), • OCD-relevancy: OCD relevant (32.4%), not OCD relevant (66.4%) Association context links, ego-dystonicity and symptoms: • 95.9% of ego-dystonic intrusions occurred without evidence • ↑ OCD symptoms, inferential confusion, OCD related beliefs and ego-dystonicity in participants having intrusions without evidence • Ego-dystonicity not related to OCD symptoms • Thoughts without evidence had a similar likelihood to be OCD-relevant (47.2%) or non-OCD-relevant (52.8%) • Thoughts with evidence were almost always non-OCD relevant (98.5%) → Much higher likelihood of thoughts without evidence being OCD-relevant than thoughts with evidence
Fradkin and Huppert (13),	**Study 1**: N = 170 non-clinical participants **Study 2**: N = 180 (OCTQ + other questionnaires) and N = 211 (OCTQ only) non-clinical participants **Study 3**: N = 126 non-clinical participants	**Study 1:** Out-of-context thoughts questionnaire including the subscales Cognitive Context, Affective Context, Self Context and Resisted Thoughts (OCTQ, 13), Obsessive-Compulsive Inventory-Revised (OCI-R; 26): total score, obsessing subscale, and checking, ordering and washing subscales utilized **Study 2**: OCTQ (final 12-item scale), OCI, Depression anxiety and stress scale (DASS-21; 27), Big Five Inventory 10 (BFI-10; 28), Mind Wandering Deliberate (MW-D) and Spontaneous (MW-S) scales (29) **Study 3**: modified version of OCTQ (provide two examples of either negative or non-negative OOCTs and answer questionnaire on the basis of thoughts of particular valence, OCI-R (26)	**Study 1:** Frequencies: 49.41% experience mostly negative out-of-context thoughts (OCT), 39.41% mostly neutral OCT, 7.05% mostly positive OCT. • Positive association between OCD symptoms (OCI-R total) and OCTQ total and subscales • OCI-R obsessions subscale sign. associated with OCTQ • Non-significant association between OCI-R subscales associated with reactive obsessions and OCTQ • Negative OOCT → more negative emotional responses **Study 2**: • Sign. positive association OCTQ and OCI-R • ↓ correlations of reactive obsessions with OCTQ than obsessing subscale (as in study 1) • Spontaneous mind wandering (MW-S) and OCTQ total and subscores: Sign. positive association • ↓ correlations between OCTQ and BFI (divergent validity) • Correlations OCTQ and OCI-R not moderated by valence. **Study 3:** • ↑ OCTQ scores (and higher OCI-Rs) predicted ↑ negative emotional responses, stronger tendencies to resist and more appraisals regarding ones personality for negative OOCTs → reduced predictability of thoughts associated with more aversiveness • Strong association between OCTQ scores for negative and non-negative OOCTs → Experience of OOCTs is independent of thoughts valence
Audet et al. (20),	N = 557 non-clinical participants	**Task:** Contextual vignettes task (CVT): 10 vignettes with different themes and two levels of evidence for their truth. Themes: aggressive, homosexual, religious/social convention, contamination, and checking). Each theme once combined with direct evidence and without direct evidence. Indicate which intrusions (from a list) would be experienced after each scenario and likelihood of having these, related distress and unwantedness from 0 to 100. **Measures**: Depression Anxiety Stress Scale 21 Item Version (DASS-21; 30); only depression subscale used); Vancouver Obsessional Compulsive Inventory (VOCI, 21), Feared Self Questionnaire (22) Inferential Confusion Questionnaire Expanded Version (ICQ-EV; 31) Obsessive Beliefs Questionnaire (32)	• All dependent variables significantly different from 0 → CVT simulates situations in which intrusions occur • Scenarios with direct evidence associated with sign. ↑ intrusions, and ↑ ratings of likelihood, distress, and unwantedness • Scenarios without direct evidence predicted OCD symptoms (predictors: number of intrusions, probability of these, distress, unwantedness) • Inferential confusion predicted likelihood, distress and unwantedness of intrusions without evidence
Llorens-Aguilar et al. (14),	N = 68 individuals with OCD	**Measures**: Obsessional Intrusive Thoughts Inventory (INPIOS; 33) frequency unwanted obsessional intrusive thoughts, images, impulses and appraisals and control strategies of these. Divided in autogenous (e.g. aggressive, sexual) and reactive (e.g. contamination). Choose most upsetting one and rate it based on emotional reactions, difficulty in controlling it and dysfunctional appraisals. Report frequency of control strategies used. **Interview:** Semi-structured interview of obsessions and intrusive thoughts in OCD and INPIOS-2^nd^ part (33): Describe intrusion constituting obsessions and intrusion, which never became obsession and other features (e.g. theme) of it. Elaborate functional consequences of these.	Frequency thoughts: • Obsessions > intrusions Thought **content** and reactions: • 1/2 autogenous, 1/2 reactive • No content differences between obsessions and intrusions • Obsessions more inconvenient than intrusions, associated with ↑ anxiety and sadness, ↑ difficult to control, associated with ↑ dysfunctional appraisals **Context**: • First occurrence obsessions: negative mood, higher stress, relevant previous stressful life event (same pattern for 1/2 the intrusions) • Most obsessions and intrusions occurred with indirect link (obs: 49.3-67.9%, in: 66.7-71.1%), some with no link (obs: 25-47%, in: 21.8-26.7%), few with direct link (obs: 3-7.1%; in: 6.7-7.9%) • Context of occurrence of obsessions significantly changed from onset to last time experienced: onset most often indirect, last occurrence indirect or no link (no context differences between first and last occurrence for intrusions) • Content and context: Onset: Autogenous and reactive obsessions and intrusions indirectly linked. Last time experienced: Autogenous obsessions and intrusions more frequently occur with an indirect link, while reactive obsessions are experienced with no link (obsessions) or indirect/no link (intrusions)

↑ = increased; ↓ = decreased; → = either "evoked/leads to" or it indicates a conclusion.

The study by Julien and colleagues (12) examined contextual aspects of intrusive thoughts in a group of individuals diagnosed with OCD (N = 33) and compared them to a control group of healthy individuals (N = 99). Participants were instructed to identify their three most disturbing intrusive thoughts and then indicate the degree to which the thought is related to the external context (direct, indirect, no link) after having received training to understand these categories. In order to explore whether the context link depends on the specific content of the intrusive thought, the authors additionally categorized them into the domains symmetry, checking, washing, impulse phobia, hoarding, rumination and schizotypy. The relationship of a thought to external context could either be direct or indirect or there is no link the external context. To exemplify the three different categories used (direct, indirect and no link), we will use the thought “I may not have extinguished my cigarette correctly” based on Julien and colleagues (12). This thought in a situation, where there is observable evidence of a cigarette still emitting smoke would be classified as having a direct link (12). A thought with an indirect link is characterized by the obsessions theme not being directly supported by clear and accurate information from the context, but still being loosely associated with it (12). For instance, the same thought in a situation when the cigarette has been extinguished and there is no information indicating that it in fact has not been extinguished well (12). In this case, a link between theme of the intrusive thought (cigarette still burning) and the context in which it occurs (seeing the cigarette) exists, but the link is indirect. Finally, no link between the content of an intrusive thought and the context of occurrence means that the intrusive thought above is detached from the external context (e.g. occurs while taking a shower) and is rather triggered by an internal narrative unrelated to the current context (34). Importantly, this three-level classification refers only to external context. When it comes to internal context, distinguishing between direct and indirect evidence can be challenging due to limited introspection and a lack of clear criteria for defining links between intrusive thoughts and internal contextual events, such as a feeling. Of note, intrusive thoughts instead of obsessions were assessed to ensure comparability between individuals with OCD and non-clinical control participants in the study. Furthermore, the study was based on the assumption that nonclinical intrusive thoughts and obsessions are similar in content and differ only with regard to their appraisal (35, 36). This study demonstrated that the link between context and intrusive thoughts differed between individuals diagnosed with OCD and unaffected control participants (12). Intrusive thoughts of non-clinical control participants were mostly directly linked to context, whereas intrusive thoughts of participants diagnosed with OCD were only indirectly linked to context. Importantly, the strength of context link differed depending on the content of the intrusive thought; for example, thoughts related to symmetry were more likely to be directly linked to a trigger within the context compared to thoughts about washing. Interestingly, on an individual level, the link between intrusive thoughts and context tended to remain consistent across all three reported intrusive thoughts (12), suggesting that the connection between context and thoughts is a stable characteristic within a person. In summary, these findings suggest that the relationship between intrusive thoughts and the external context is diminished in individuals with OCD.

More recently, Llorens-Aguilar and colleagues (14) examined whether the link to context differed between intrusive thoughts and obsessions and whether this link changed over the course of the disorder. During an interview procedure, 68 individuals with OCD were asked to report their most upsetting obsession and intrusive thought, detailing how these thoughts occurred at different time points (14). Then, these descriptions were categorized by experts according to the link with external context using the three levels described above (direct, indirect, no link). It was found that during the first occurrence, both obsessions and intrusive thoughts were most commonly indirectly linked to the context. This typically occurred when individuals experienced negative mood and increased stress levels compared to their usual state. Moreover, the connection between thought and external context diminished over time for obsessions but not intrusive thoughts. Consequently, at the time point of the last experience, obsessions were equally likely to occur with indirect as they were with no link to context. In contrast, the link between intrusive thoughts and context remained stable over time. The authors argue that in the course of OCD, obsessions become more automatic and detached from context, which might be based on deficient learning (14). Compared to intrusive thoughts, obsessions are characterized by the repetition and increased automaticity of thoughts, coupled with an increasing detachment from context over time, which is not a defining feature of intrusive thoughts. However, the results of the two so far reported studies indicate that this detachment from context is already apparent in intrusive thoughts, which were found to have an indirect connection to the context in OCD patients (14), while being directly linked to the context in healthy individuals (12). Furthermore, the study examined the context links with regard to thought content. As proposed by Lee and Kwon (16), thoughts with sexual, aggressive, or immoral contents were classified as autogenous obsessions and thoughts on contamination, mistakes, accidents, asymmetry or loss are considered as reactive obsessions. Importantly, Lee and Kwon (16) suggested that autogenous thoughts or obsessions are less likely to depend on external evoking stimuli than reactive thoughts or obsessions. However, Llorens-Aguilar and colleagues (14) found that intrusive thoughts and obsessions that would be classified as autogenous according to Lee and Kwon (16), i.e. involving sexual and aggressive themes, occur with indirect evidence, while reactive thoughts and obsessions occur with no link to the context (14). Contrary to the exact classification of themes with context links in the initial proposal by Lee and Kwon (16), this partly aligns with Julien and colleagues (12). In sum, this research indicates that the thematic nature of intrusive thoughts and obsessions may play a crucial role in determining the connection between thoughts and context.

In a non-clinical study, Audet and colleagues (19) asked N = 248 non-clinical participants to report an intrusive thought they experienced and describe the context in which it occurred. Clinicians rated these intrusive thoughts as either directly linked to context or not linked to context. While intrusive thoughts with no link to context predicted OCD symptoms as measured by the Vancouver Obsessional Compulsive Inventory (VOCI, 24), intrusive thoughts that were linked to the context did not (19). Additionally emphasizing the altered role of context in OCD, intrusive thoughts with no link to context correlated with increased scores on inferential confusion measures (19). Inferential confusion is a metacognitive reasoning process, in which an imagined possibility is mixed up with an existing probability (37). The correlation indicates that conclusions may be reached without adequate consideration of contextual evidence (19), potentially connected with increased out-of-context thoughts. Interestingly, ego-dystonicity of a thought, which is characteristic for OCD, was not able to predict OCD symptoms (19). Ego-dystonic thoughts contradict the persons moral values or intentions, and are therefore perceived as invasive and irrational, and not linked to the internal context of a person (19, 38). This suggests that for OCD symptoms, intrusive thoughts might be independent of external context rather than internal context (ego-dystonicity), suggesting a smaller role for ego-dystonicity than traditionally thought (19). However, the authors note that the absence of identifiable triggers for the intrusive thoughts might alternatively reflect impaired introspection and access to mental states as part of the disorder (19, 39) instead of a decoupling of context and OCD-related thoughts. Because of the link with inferential confusion, the importance of therapeutically addressing subjective reasoning processes (metacognitive processes) that might be biased in OCD is emphasized (19).

In three other non-clinical studies, Fradkin and Huppert (13) examined non-clinical samples (N = 599) by means of the “Out-of-Context Thoughts Questionnaire (OCTQ)” which was developed by the authors (13). As a result, increased thoughts with no link to context were significantly associated with OCD symptoms on the Obsessive-Compulsive Inventory-Revised, OCI-R (13, 26). The OCTQ examines the decoupling of thoughts from context along four domains: Cognitive context, where thoughts are unexpected relative to previous ones; Affective context, thoughts unexpected relative to current emotions; Self context, thoughts unexpected relative to the own self-concept; Resisted thoughts, thoughts unexpected despite the efforts to control them (13). While the total score on the OCI-R and the obsessions, checking and neutralizing subscales were consistently significantly associated with increased thoughts with no link to context, findings for the hoarding and ordering subscale of the OCI-R were mixed in the two studies assessing this. Interestingly, the washing subscale of the OCI-R was not associated with increased thoughts with no link to context. Generally, the correlations of the subscales that are associated with reactive obsessions with OCTQ were lower than for the autogenous obsessions subscale. This is consistent with the theory of Lee and Kwon (16) claiming that washing as a reactive obsession possesses a higher likelihood of being triggered directly, and is in line with Julien and colleagues (12) who found that reactive obsessions (in their study hoarding and symmetry) occur closely linked to context. However, it remains unclear why the checking subscale, which is also considered a reactive obsession, significantly correlated with OCTQ in the two studies. Importantly, as the authors note, the subscales of the OCI-R that are associated with reactive obsessions do not measure reactive obsessions as such, but instead compulsive behaviors connected to these, which further explains the differential association with thoughts not linked to context (13). Additionally, the authors suggest that the decoupling of thoughts from the current context makes obsessions unpredictable and thus highly aversive and uncontrollable which increases the probability that the thoughts reappear explaining the vicious circle of obsessions (13). However, generalizability of results is limited since only unaffected individuals were included and self-reports were used to assess thoughts with no link to context.

As the first study investigating the role of context in OCD symptoms experimentally instead of self-reports (20), utilized the contextual vignettes task (CVT) in unaffected participants (N = 557). In the task, different scenarios of various OCD themes are described, such as aggression or contamination. The scenarios in the stories are either directly linked to the context (i.e., provide direct evidence for the reality of a subsequent thought) or unlinked to the context (lack direct evidence for the reality of the thought). An exemplary scenario of the aggression theme without link to context is a story about the reader watching a movie about a psychopath who kills his family, but appeared normal to others before. During the movie, the readers family arrives home and a suggested potential intrusive thought is that the reader might harm his/her family. After reading the stories, participants were shown potential intrusive thoughts and selected the ones that could occur to them in this situation, the likelihood of these and the corresponding distress and unwantedness. As a result, corresponding with other studies (10, 13), it was demonstrated that individuals with an increased likelihood of reporting intrusive thoughts with no link to context showed higher OCD symptom scores (VOCI, 24). These individuals additionally showed increased levels of ego-dystonicity and judged their intrusive thoughts as more repugnant (20). Furthermore, similar to Audet et al. (19), inferential confusion predicted likelihood, distress and unwantedness of intrusive thoughts (20), emphasizing the strong association of an altered role of context with other OCD phenomena.

Overall, the described studies suggest that OCD symptoms (pathological obsessions) are less connected to both, external (12, 14, 19, 20) and internal (13) context. Importantly, the degree of thought-context decoupling distinguishes intrusive thoughts in unaffected individuals from obsessions in OCD, where there are reduced relations to context in the latter group (12). In line, studies in unaffected individuals demonstrated that intrusive thoughts with reduced relations to context predict OCD symptoms (13, 19, 20), while intrusive thoughts linked to context do not (19, 20). However, due to mixed evidence, the degree to which thoughts in OCD relate to context is still unclear i.e. whether obsessions are more likely to be indirectly linked or not linked with context at all (12, 14). It is possible that this depends on the stage of the disorder with a more advanced stage resulting in a reduced coupling of thoughts with context as a function of increased automaticity of obsessive thoughts. This interpretation is supported by evidence from Llorens-Aguilar et al. (14) who showed that obsessions increasingly decouple from the current context. Furthermore, evidence indicates that the degree to which obsessions in OCD are linked to context relates to the content of obsessions, i.e. different topics of obsessions are not affected by decontextualization equally (12, 14). Overall, there is strong evidence for alterations in the thought-context coupling in OCD.

## 3 Discussion

The literature presented suggests a diminished link of obsessions to context in OCD, particularly in the sense that both intrusive and obsessive thoughts seem to have weaker connections to external and internal contextual factors (12–14, 19, 20). Generally, obsessions tend to have indirect or no links with context, and this tendency appears to intensify with the progression of symptoms (12, 14). The degree of thought-context decoupling in OCD seems to depend on the specific theme of obsessions (12, 14). Differences in contextual links have already been considered in the distinction between autogenous and reactive obsessions (16). Reactive obsessions were assumed to be related to the external context, whereas autogenous obsessions were thought to manifest spontaneously without any direct links to the external context. However, these relationships did not replicate in the studies investigated here. For example, Julien et al. (12) found that in line with the assumption of Lee and Kwon (16), symmetry was more directly linked to context. Surprisingly, contamination, which was proposed as a reactive obsession and might be anticipated to have a similar direct link to context, was found to be more indirectly linked. Moreover, Llorens-Aguilar et al. (14) demonstrated that autogenous obsessions are more related to the external context than reactive ones. In sum, while some themes occur less linked with context than others, more research is needed to figure out which contents should be grouped together to represent thoughts more closely related to context and those less related to context. Potentially, new labels other than autogenous and reactive should be considered to prevent confusion with the old categories.

Obsessive thoughts that occur unrelated to the context play a significant role in OCD, but it is not clear how these thoughts contribute to OCD. One perspective suggests that when these obsessions arise unexpectedly and out-of-context, they may be perceived as threatening (13). This idea is supported by research indicating that negative thoughts are perceived as more aversive when they occur out-of-context (13). This perception of heightened threat may add to the aversiveness of the thought itself and then further increase the tendency to engage in efforts to control their thoughts (13, 40, 41). Paradoxically, these efforts to control thoughts could increase the salience and significance attributed to out-of-context thoughts, further characterizing them as obsessions (13).

In addition, Fradkin and colleagues (13) have suggested that individuals with OCD have altered meta-cognitive beliefs about the predictability of normal thoughts, leading them to magnify the significance of out-of-context thoughts. This in turn, prompts increased attempts to control these thoughts and eventually increase their salience (13). However, it is unlikely that altered meta-cognitive beliefs are the only cause of out-of-context-thoughts. Clinicians have identified thought-context decoupling (19), experimental evidence has also established a link between out-of-context thoughts and OCD (20).

We propose the term “decontextualization of thoughts” to describe the phenomenon that obsessions lose their connection with context. Evidence suggests that decontextualization of thoughts occurs gradually during the development of OCD disorder (14), which is potentially contributing to the severity of symptoms. This process may by driven by increased automaticity of thoughts due to alterations in learning in OCD. Furthermore, a reduced sense of Agency (SoA) has been observed in OCD (42). It has been shown that decontextualization is associated with a decreased SoA over thoughts, which is defined as the feeling that ones own thoughts originate from oneself (43) as well as increased distrust of the senses (19, 44). More specifically, individuals with OCD experience themselves as less autonomous and more passive in the thought-generation process (43). As a result, they may not experience context as salient and clear as unaffected participants, making it challenging to adequately associate thoughts with context. If individual with OCD perceive the context less clearly, it may lead to a perception of infinite possibilities for actions in a situation and therefore thoughts might seem less predictable. Another perspective suggests that alterations in the ability to process contexts (“context processing”), could explain the detachment of obsessions from their context. Similar alterations have been observed for mental disorders like schizophrenia and post-traumatic stress disorder (45–49). Context processing is one part of working memory that encompasses all processes that allow for on-line maintenance and manipulation of contextual information (such as environmental stimuli, instructions or goals) (45). This enables adaptive responses to stimuli in the presence of distraction (45–47, 50, 51). Alterations in context processing may represent a transdiagnostic phenotype affecting psychopathology, such as OCD. Studies in OCD have shown an intact ability to detect context changes, yet these changes are often disregarded in subsequent actions (52). Fradkin and colleagues (53) on the other hand found deficits in recognizing shifts in cue-outcome contingencies (i.e. context changes) in individuals with OCD. Hence, it remains unclear whether individuals with OCD have difficulties in perceiving and recognizing context changes or whether they are only impaired in applying this contextual knowledge to guide appropriate behavior.

### 3.1 Limitations

Despite a significant body of evidence demonstrating an association between decontextualization and OCD in various studies, it is essential to acknowledge that the majority of studies have utilized non-clinical participants (13, 19, 20). In order to generalize findings to OCD, it is crucial to incorporate studies involving individuals diagnosed with OCD, such as those conducted by Julien and colleagues (12) and Llorens-Aguilar et al. (14). Another limitation of prior studies is that the majority relied on self-reports, often with a retrospective element (12–14, 19). Even studies that relied on expert ratings were based on information provided by participants (14, 19). However, participants may inadvertently omit information indicating a close link with context, either due to a lack of awareness of its relevance or constraints on their introspection. Another limitation to consider is the broad and multifaceted nature of the term context compassing various aspects such as mood, life events experienced or the current action (14). Therefore, establishing a hierarchy to determine the significance of each aspect when linking it to a thought is crucial. For instance, deciding how to classify the link between a thought and context, when the current activity is unrelated, but the mood matches the thought. Without such a hierarchy, it is challenging to compare context links across participants and studies. It is important to note that the presented evidence regarding the altered link between obsessions and context is based on single studies rather than meta-analyses, and therefore has to be interpreted with caution. Nevertheless, albeit variations in methodologies, such as self-report based vs. experimental manipulation, these studies generally arrive at similar conclusions.

### 3.2 Decontextualization of behavior

Elevated decontextualization in OCD is not restricted to obsessive thoughts, but may also extend to compulsive behaviors. OCD is characterized by an increased reliance on habitual behaviors, which remain resistant to changes in context, including changes in situations or personal goals (54, 55). Thereby, habitual behavior in OCD is context-independent and persists despite changes in context. In fact, (symptomatic) compulsive behaviors in OCD rely on the habitual system, rendering them highly automatic (56), rigid, inflexible and difficult to stop (55, 57). Notably, habitual behavior is often strongly cue-driven, while the context and other aspects of the situation are neglected. In support, Gillan and colleagues (55) demonstrated an increased tendency for habitual behaviors as opposed to goal-directed behavior in OCD, and this tendency is correlated with OCD symptom severity. Furthermore, Gillan and colleagues (57) subsequently showed that individuals with OCD encounter difficulties in unlearning avoidance habits that were established based on a conditioned warning stimulus that had previously prevented them from harm. This evidence indicates an excessive focus on cues in OCD, often at the expense of not responding to (changes in) context. In accordance, previous studies have shown that control behavior in OCD is less influenced by external error signals (58) and the adjustment of behavior in OCD is less influenced by external feedback (59). In line with these findings, a classic Pavlovian-to-instrumental transfer task (PIT), which investigates the effect of Pavlovian conditioned stimuli on instrumental behavior, revealed that individuals with OCD are less able to use contextual information from the PIT cues to adapt their behavior (60). Collectively, these studies suggest that contextual information play a less significant role in behavior selection in OCD, resulting in decontextualized behavior.

As cognitive flexibility is required for goal-directed behavior (61), over-reliance on habitual, context-independent behavior can further be assessed by means of cognitive flexibility tasks. Cognitive flexibility is a fundamental executive function characterized by the capacity to change behavior or cognitive strategies when faced with new challenges, rules, environments or priorities (i.e. contexts) (61, 62). This ability can be evaluated through cognitive flexibility tasks such as the Wisconsin Card Sorting Test (WCST) (63), reversal learning paradigms (59) and set shifting tasks (64–66). Impairments in both cognitive flexibility (i.e. cognitive inflexibility) and behavioral flexibility are typically observed in OCD (67–70). As impaired cognitive flexibility has also been found in unaffected first-degree relatives, it has been considered a risk marker or endophenotype of OCD (71). In line, a meta-analysis revealed that cognitive flexibility is strongly affected in OCD (Cohens d = .517) (72). Interestingly, there are also examples where cognitive flexibility in OCD is superior to that of nonclinical control participants specifically when a previously inhibited mental set becomes relevant again (73). This seemingly paradoxical finding has been interpreted as a deficit in adapting to novel task rules, in contrast to better performance when dealing with familiar task rules (73). More generally, recent agent-based simulations have demonstrated that increased habitual tendencies (“strong habit learner”), which also overlap with the clinical picture of OCD (74), compromise the ability to recognize changes in contexts on a reversal learning task (75). In addition, alterations of economic decision making have been observed in OCD and it would also be interesting to investigate the effects of context on formal parameters of economic decision-making frameworks (e.g. 76–79).

### 3.3 Neural correlates of context processing

At the neural level, the hippocampus plays an important role in representing contextual information and is necessary for contextual learning (80, 81). Alterations in hippocampal areas and hippocampal-prefrontal-thalamic circuitries are suggested to underlie the ability to derive contextual inferences, playing a central role in the psychopathology of PTSD (48). Based on accumulating evidence for alterations in context processing in OCD, it is likely that changes in the network may also be evident in OCD, and contribute to the clinical picture of such patients. Indeed, there is evidence that hippocampal volume may be altered in OCD (82–85). Interestingly, reduced hippocampal volume has been shown in individuals who score high on ordering and checking symptoms (82). In summary, there are only few studies investigating hippocampal changes and it would be interesting to examine how hippocampal structure and function in OCD relate to context processing.

Overall, laboratory-based studies indicate a pivotal role of impaired cognitive flexibility (68–72, 86), increased habitual behavior (55, 57) and altered decision-making in OCD (76–79). In general, there is evidence for decontextualization in OCD, affecting obsessive thoughts and compulsive behavior. This is evident in the limited ability of individuals with OCD to adapt their behavior to changing contextual information (57, 69), leading to a reduction in adaptive goal-directed behavior. Alterations in context processing in OCD may be related to changes in hippocampus volume (82–85). However, while the link between decontextualization and habitual behavior as well as cognitive inflexibility in OCD is plausible, so far, no studies directly explored the role of context in compulsive behavior. As a result, the following discussion of the implications will focus on decontextualization of obsessions.

### 3.4 Future directions

Given the finding that obsessions in OCD tend to decontextualize, potentially contributing to the disorder (14), an important question arises: Can this progress be counteracted, perhaps through therapeutic interventions? A secondary consultation would be to explore whether such interventions might lead to an improvement in symptoms. In particular, already in the early stages of the disorder, potential overgeneralization of obsessions should be addressed, i.e., it should be examined whether a particular obsession is justified in a particular context, based on the strength of the context link (direct, indirect or no link) with the original trigger of the obsession. The crucial question would be how to increase salience of context, both internal and external, in a way that minimizes the occurrence of decontextualized thoughts by reducing them proactively. Of note, such an approach would require an understanding of other predisposing factors that could indicate a potential progression towards an OCD-like pathological state, such as increased distress, which has been associated with the emergence of decontextualized thoughts in OCD (12).

An avenue for future research lies in understanding why seemingly similar intrusive thoughts and obsessions diverge progressively over time with regard to their link to context and how this development can be predicted (14). Secondly, it should be investigated whether this development is related to OCD severity. Additionally, it should be deciphered how the distinct contents of obsessions relate to decontextualization. Reliable insights from context measures, such as out-of-context thoughts (13), could potentially serve as predictive indicators for the development of OCD and inform the creation of new treatment approaches. Furthermore, additional experimental clinical studies are needed to rule out meta-cognitive beliefs as a contributing factor to decontextualization in OCD.

## 4 Conclusion

Taken together, we suggest that obsessions and potentially compulsions in OCD are decontextualized (12, 14, 19, 20, 57). The reduced reliance on contextual information to guide thoughts and behaviors in OCD may be associated with the reduced adaptability and flexibility often observed in the condition (68–70, 87). Decontextualized thoughts and behaviors in OCD might lead to an exaggerated emphasis on the significance of cues related to symptoms (88) and result in overgeneralization across contexts.

## Author contributions

FW: Conceptualization, Investigation, Writing – original draft, Writing – review & editing. KS: Writing – review & editing. TE: Supervision, Writing – review & editing.
